# Design, synthesis, and application in OFET of a small molecule based on π-expanded fused diketopyrrolopyrrole

**DOI:** 10.3389/fchem.2023.1280816

**Published:** 2023-10-09

**Authors:** Jianhui Li, Zhuoting Ji, Aihua He, Haichang Zhang

**Affiliations:** Key Laboratory of Rubber-Plastics of Ministry of Education/Shandong Province (QUST), School of Polymer Science and Engineering, Qingdao University of Science and Technology, Qingdao, China

**Keywords:** organic field effect transistor (OFET), p-type, charge transport mobility, semiconductor, conjugated materials, DPP

## Abstract

Diketopyrrolopyrrole (DPP) and its derivatives, as electron deficient units, are widely used as building blocks in organic field-effect transistors, obtaining high performance. However, further modification of the DPP structure is crucial for the development of organic semiconductors. In this work, an FDPP is synthesized and characterized. The results show that FDPP exhibited not only a good planar core structure with a good conjugation system, but also strong aggregation in the solid state. As a consequence, FDPP presents p-type behavior with a hole mobility of ∼9.7 × 10^−3^ cm^2^ V^−1^ s^−1^. This study suggests that FDPP is a promising electron deficient unit for high performance semiconductors.

## 1 Introduction

In the past few years, organic-field effect transistors (OFETs) have received significant attention by the academic society due to their multiple advantages and potential applications, such as solution processing, lightweight, large area, and compatibility with flexible substrates. (Surya et al., 2019; Sonalin et al., 2020; Zhu et al., 2020; Chen et al., 2022; Shibuya et al., 2022; Zhang et al., 2022; Mooney et al., 2023). Rapid progress in the field of OFETs has led to a renaissance in the chemistry of dye molecules, compelling scientists to revitalize well-known and relatively new, so-called high-performance pigments such as diketopyrrolopyrroles (DPPs), (Zhang et al., 2020a), isoindigo, (Zhang et al., 2020b), quinacridones, (Yuvaraja et al., 2020), and naphthodifuranone (Tao et al., 2022). Thus, an increasing number of researchers have focused their efforts on the development of high-performance pigments with high charge transport mobility (Ali and Siddiqui, 2022). Among these, diketopyrrolopyrrole, indigo, and their derivatives, as electron deficient building bulks, are very popular and promising for the design high-performance semiconductors (Shaker et al., 2020; Li et al., 2022; Lu et al., 2022).

For organic semiconductors, the charge transport mobility, open-circuit voltage (V_oc_), and on/off current ratio are three crucial factors. DPP represents a key structural unit in an important class of red pigments with deep color, which were commercialized in the 1980s (Cui et al., 2022). It was shown that DPP-containing conjugated materials exhibit high charge transport mobility and excellent photovoltaic properties (Rabindranath et al., 2006; Tung et al., 2016; Balambiga et al., 2022). Charge transport in the semiconductor should not only occur in single molecules, but also across neighboring molecules. Thus, charge transport is divided into inter- and intracharge transport. For small molecules, due to short effective π-electron delocalization and good molecular packing, intercharge transport plays a key role in small molecule–based semiconductors (Hofkens et al., 1998; Guo et al., 2009; Yang et al., 2013).

π-conjugation extension and good planar structures are not only effective for intra-charge transport, but also benefit molecular packing and π-π overlap (Devibala et al., 2021; Feriancová et al., 2022). A new small molecule, based on π-expanded fused DPP, namely, FDPP, received our attention (Grzybowski et al., 2012). To the best of our knowledge, only a few articles have reported FDPP-based polymers and applied them in OFETs that present ambipolar properties with excellent ambipolar semiconducting properties under ambient conditions, reaching 2.23 and 1.08 cm^2^ V^−1^ s^−1^ for the n- and p-channels, respectively. However, semiconductor properties based on FDPP are completely unknown. In this work, FDPP is synthesized according to the literature and is applied in an OFET as the p-type semiconductor for the first time, presenting a hole mobility of approximately 1.3 × 10^−3^ cm^2^ V^−1^ s^−1^. In addition, the optical and electrochemical properties, as well as computation results, are investigated.

## 2 Results and discussion

### 2.1 Optical properties

**FIGURE 1:**
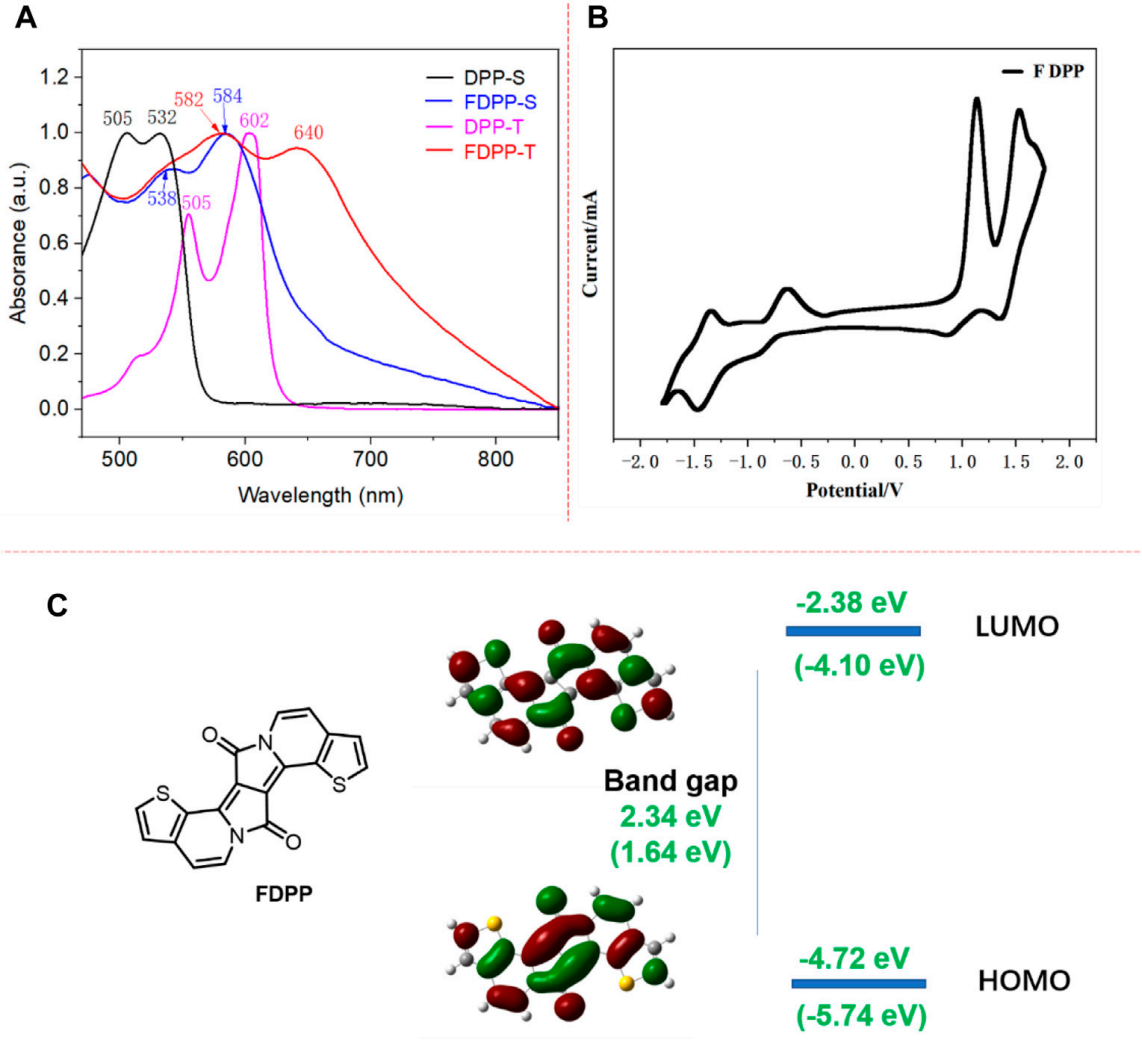
**(A)** UV/vis absorption spectra of DPP and FDPP in solution and thin film state; **(B)** cyclic voltammetry spectrum of FDPP; and **(C)** computational calculations of FDPP obtained at the B3LYP/6-31G* level.

For the purpose of evaluating the optical properties, UV/vis spectroscopies of FDPP as well as of the DPP precursor are performed in dichloromethane solution and in the thin film state. The corresponding optical data are presented in Figure 1A. The alkylated DPP presents pink and purple colors in the solution and thin film state, respectively, while FDPP exhibits a pink (solution state) and blue color (thin film state). The absorption spectrum of the DPP precursor in dichloromethane exhibited a strong absorption maximum (λ_abs.max_) at 532 nm, with a band peak at 505 nm. The extinction coefficient at 532 nm was 4.1×10^4^ L mol^−1^ cm^−1^. Compared to the DPP precursor, the λ_abs.max_ of fused FDPP is shifted to 584 and 538 nm, which is red-shifted by 52 nm. The large red-shift is ascribed to fusing of the thiophene ring and the DPP core, resulting in improved planarity of the molecule backbone, as well as π-conjugation extension, which is advantageous for delocalized intramolecular π-orbitals and thus increased, efficient conjugation lengths (Dai et al., 2022). In the thin film state, both molecules presented a red-shift (70 nm for DPP and 66 nm for FDPP) in the optical absorption spectra compared to the solution state. The large red-shift indicates strong aggregation. In addition, in the long-wavelength absorption range, the bathochromic shift seems stronger for FDPP (around 150 nm). This observation indicates that FDPP presented a stronger π-π interaction as well as long-range ordered packing. This could be ascribed to the fact that FDPP presents a more planar structure core compared to DPP, which is advantageous for molecular packing as well as for π-π overlap. According to the onset of absorption, the optical band gap is calculated to be 1.55 eV for FDPP.

### 2.2 Electrochemical properties

The electrochemical properties of FDPP are investigated through cyclic voltammetry. As shown in Figure 1B, FDPP exhibited quasi-reversible reduction and oxidation curves. The reductive cycle showed two quasi-reversible cathodic waves at −0.63/−0.90 V and −1.37/−1.45 V. These quasi-reversible cathodic waves might have originated from the reduction of the DPP core from quinoid to a benzoid anion and dianion structures (Zhang et al., 2013). During the positive scans, a quasi-reversible redox wave was observed at 1.13/0.88 V and 1.55 + 1.63/1.36 V. The oxidative cycle exhibited three anodic waves at +1.13, +1.55, and +1.63 V, which are reverted at +1.36 and +0.88 V. Based on the onset of oxidation and reduction potentials, the HOMO/LUMO energy levels as well as the electrochemical band gap of the FDPP are estimated. As shown in Figure 1B, the onset of oxidation and reduction occurred for FDPP at 0.94 and −0.70 eV, respectively, based on which the HOMO and LUMO energy levels were calculated to be −5.74 eV and −4.10 eV, respectively. The HOMO energy level of FDPP is lower than the oxidation threshold of air, i.e., −5.27 eV, indicating the good stability of the material in air. According to the HOMO/LUMO energy levels, the electrochemical band gap is calculated to be 1.64 eV. The electrochemical band gap is slightly larger compared to the optical, which might be due to an interfacial barrier for charge injection (Zhu et al., 2009).

### 2.3 Computation

For the purpose of investigating the frontier molecular orbital features, the backbone configuration, and the HOMO/LUMO energy levels of FDPP, computational calculations were conducted using density-functional theory (DFT) at the B3LYP/6-31 (d,p) level of FDPP. As shown in Figure 1C, the core of FDPP is completely planar, which is in agreement with the optical results, while there is an approximately 10° angle between the thiophene and the DPP core (Naik et al., 2012). The electronic cloud distributions of the HOMO are mainly located at the DPP core, while the LUMO electronic cloud is dispersed over the whole of the FDPP. This observation indicates that if the FDPP is excited, electron transfer from the DPP core to the whole FDPP could take place, which means that FDPP has a weak intramolecular charge transfer (ICT) effect. The calculated HOMO/LUMO levels of the FDPP are −4.72 and −2.38 eV, resulting in a band gap of 2.34 eV. This value is larger than the electrochemical results, which is ascribed to the fact that the CV measurements were carried out on thin films of FDPP with strong intermolecular interactions, whereas the calculated results were based on a single FDPP molecule without intermolecular interactions.

### 2.4 OFET

**FIGURE 2:**
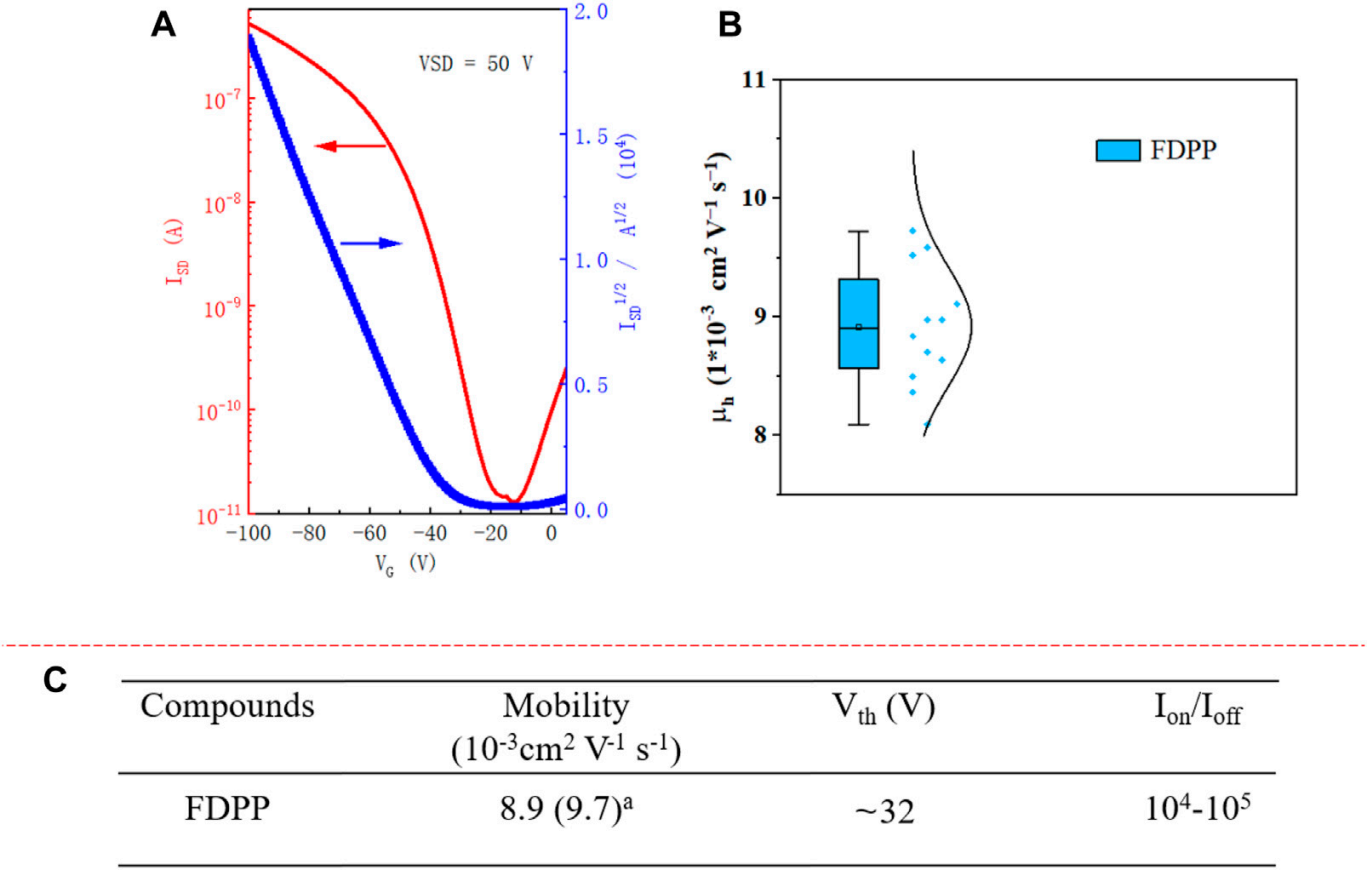
**(A)** Characteristics of FDPP OFET devices; **(B)** hole transport mobility obtained from 12 different devices; **(C)** hole mobilities (µ_h_), threshold voltages (V_Th_), and on/off ratios (I_on_/I_off_) of the FDPP-based FET device. The mobility is provided in average (highest)^a^ form, with the performance based on 12 different FETs. Mobility was extracted by fitting the linear part of the plot of I_DS_^1/2^ *versus* V_G_ using the equation I_DS_ = C_i_µ(V_G_-V_Th_)^2^ W/2L. ^a^ hole mobility.

The charge transport performance of FDPP was evaluated in bottom-gate, bottom-contact (BGBC) OFET devices on an n-type silicon wafer with a 300 nm layer of SiO_2_ as a dielectric material. After cleaning the substrate with piranha solution (H_2_SO_4_/H_2_O_2_, 3/1 v/v), deionized water, and acetone, it was immersed in OTS solution (5% in toluene) at room temperature overnight in an Ar-filled glove box. Subsequently, gold (30 nm) source (S) and drain (D) electrodes were deposited on the wafer through a shadow mask. The channel width was 1,000 µm and the distance between S and D electrode was 50 µm. A 5 mg/mL FDPP solution (in chloroform) was dropped on the surface of the substrate using a syringe (with PTFE filter, 0.45 μm) in an Ar-filled glove box. After 0.5 h, the temperature was increased to 45^o^C for 1 h on a hotplate. This mild heating is helpful for removing the solvents. The small molecule OFET device was prepared in an Ar-filled glovebox and characterized under vacuum condition. As shown in Figure 2A, FDPP exhibited p-type behavior, with an average hole mobility of 8.9 × 10^−3^ cm^2^ V^−1^ s^−1^ (highest μ_h_ of 9.7 × 10^−3^ cm^2^ V^−1^ s^−1^), while the non-fused precursor alkylated DPP device exhibited a hole mobility of only approximately 4 × 10^−3^ cm^2^ V^−1^ s^−1^ (Courtemanche et al., 2015). Compared to the alkylated DPP precursor, the hole transport mobility of the fused FDPP is enhanced more than double. This could be ascribed to the facts that 1), the planar core structure and the π-conjugation extension result in large efficient conjugation lengths in FDPP, which are advantageous for hole transport and 2), strong aggregation and good molecular packing are advantageous for hole transport between neighboring molecules. The current on/off ratio (I_on_/I_off_) of the device is ∼10^5^, while the threshold voltage is ∼32 V.

## 3 Conclusion

In this work, a new π-conjugation extended fused DPP, namely, FDPP, was synthesized and characterized. In the thin film state, FDPP presented a large red-shift and strong absorption in the long wavelength range compared to the solution state, indicating that FDPP exhibited strong aggregation and good molecular packing in the solid state, which are advantageous for intercharge transport. The computation results showed that the FDPP core exhibited a completely planar structure. In addition, compared to the DPP core, the core of FDPP exhibited π-conjugation extension behavior, with a weak ICT effect. This observation is beneficial for charge transport within the FDPP molecules. As a consequence, the FDPP based semiconductor presents p-type behavior, with a hole mobility of up to 9.7 × 10^−3^ cm^2^ V^−1^ s^−1^ and an I_on_/I_off_ ratio of approximately 10^5^. This study suggested the great potential of FDPP-type chromophores in constructing novel organic semiconductors.

## Data Availability

The original contributions presented in the study are included in the article/supplementary material, further inquiries can be directed to the corresponding author.
